# A Plea for Harm Reduction Policing Involving People Who Use Drugs

**DOI:** 10.15171/ijhpm.2018.29

**Published:** 2018-04-08

**Authors:** Ehsan Jozaghi, Lorna Bird

**Affiliations:** ^1^The British Columbia Centre for Disease Control, Vancouver, BC, Canada.; ^2^The School of Population and Public Health, Faculty of Medicine, University of British Columbia, Vancouver, BC, Canada.; ^3^Vancouver Area Network of Drug Users, Vancouver, BC, Canada.; ^4^Sex Workers United Against Violence, Vancouver, BC, Canada.

## Dear Editor,

Global cases of opioid-related fatalities have reached historic proportions.^1^ Some North American jurisdictions have seen overdose fatality cases that far outpace motor vehicle accidents.^2,3^ While opioid use through intravenous methods have contributed to the majority of drug overdose fatalities,^4^ in their recently published paper, Klar et al^5^ raise an important issue concerning the frequency of opioid overdoses attributed to smoking. What is more concerning is that there are increasing reports of overdose fatalities attributed to insufflation or ingestion of illegal drugs containing fentanyl derivatives.^4,6^

## Ways Forward

The current number of global opioid overdose fatalities continues to increase as synthetic opioids are increasingly manufactured illegally in homemade labs.^7^ Some cities in the province of British Columbia (B.C.), Canada, have created innovative ways of tackling some of the challenges posed by people who may consume illegal drugs via smoking, ingestion, or insufflation.^8,9^ The city of Vancouver, BC, is making further progress by transitioning away from providing services via standalone injection facilities and city of Surrey, BC has also begun offering supervised spaces for people who use drugs (PWUD) via ingestion or insufflation.^8,9^

## Harm Reduction Policing

Despite the noted advancements, overdose fatalities continue to occur in record numbers.^10^ We recommend a paradigm shift in the current legal and social framework that recognizes illegal drug addiction as a physiological medical condition rather than a moral relapse. The current framework is isolating and destroys the already-fragile social network of PWUD due to fears of stigmatization and criminalization.^11^ For example, in 2017, 90% of overdose fatalities in the province of B.C. occurred indoors, and in most cases, in isolation.^10^ There is evidence to suggest that harm reduction approaches to law enforcement could offer a variety of avenues to reduce morbidity and mortality.^11,12^ When law enforcement is implemented through a harm reduction philosophy, low level crime related activities such as prostitution and small drug offences are dealt with through health care models.^11,12^ Simultaneously, this would contribute to the expansion of evidence-based harm reduction avenues, improve public safety, and reduce overdose fatalities involving PWUD, especially recreational users or new PWUD who are more likely to smoke, ingest or insufflate.^13^

## Acknowledgements

We would like to thank Caimen Yen, Jane A. Buxton, and Aiyanas Ormond for their assistance on this letter. We also would like to thank the board members of VANDU for reading the revised draft of this letter and providing feedback and corrections. The contribution by EJ was supported by the Canadian Institutes of Health Research (CIHR) Postdoctoral Fellowship (201511MFE-358449-223266). We are also grateful to the anonymous reviewer who enhanced and improved the quality of this letter.

## Ethical issues

Not applicable.

## Competing interests

The views expressed by EJ and LB in this letter are those of the authors, and they may not necessarily express the views of the Canadian Institutes of Health Research (CIHR), Ottawa, ON, Canada or the British Columbia Centre for Disease Control (BCCDC), Vancouver, BC, Canada. The CIHR and the BCCDC had no influence on the direction, scope, opinion, view point or the writing of this letter.

## Authors’ contributions

Both authors contributed equally to the writing of this letter.

## Authors’ affiliations

^1^The British Columbia Centre for Disease Control, Vancouver, BC, Canada. ^2^The School of Population and Public Health, Faculty of Medicine, University of British Columbia, Vancouver, BC, Canada. ^3^Vancouver Area Network of Drug Users, Vancouver, BC, Canada. ^4^Sex Workers United Against Violence, Vancouver, BC, Canada.
